# Shape‐Controlled, Self‐Wrapped Carbon Nanotube 3D Electronics

**DOI:** 10.1002/advs.201500103

**Published:** 2015-06-01

**Authors:** Huiliang Wang, Yanming Wang, Benjamin C.‐K. Tee, Kwanpyo Kim, Jeffrey Lopez, Wei Cai, Zhenan Bao

**Affiliations:** ^1^ Department of Materials Science and EngineeringStanford University496 Lomita MallStanfordCA94305USA; ^2^ Department of Electrical EngineeringStanford University350 Serra MallStanfordCA94305USA; ^3^ Department of Chemical EngineeringStanford University443 Via OrtegaStanfordCA94305USA; ^4^ Department of Mechanical EngineeringStanford University440 Escondido MallStanfordCA94305USA

**Keywords:** 3D electronics, carbon nanotubes, self‐wrapped, shape‐controlled, shape memory polymer

## Abstract

The mechanical flexibility and structural softness of ultrathin devices based on organic thin films and low‐dimensional nanomaterials have enabled a wide range of applications including flexible display, artificial skin, and health monitoring devices. However, both living systems and inanimate systems that are encountered in daily lives are all 3D. It is therefore desirable to either create freestanding electronics in a 3D form or to incorporate electronics onto 3D objects. Here, a technique is reported to utilize shape‐memory polymers together with carbon nanotube flexible electronics to achieve this goal. Temperature‐assisted shape control of these freestanding electronics in a programmable manner is demonstrated, with theoretical analysis for understanding the shape evolution. The shape control process can be executed with prepatterned heaters, desirable for 3D shape formation in an enclosed environment. The incorporation of carbon nanotube transistors, gas sensors, temperature sensors, and memory devices that are capable of self‐wrapping onto any irregular shaped‐objects without degradations in device performance is demonstrated.

## 1 Introduction

This is an open access article under the terms of the Creative Commons Attribution License, which permits use, distribution and reproduction in any medium, provided the original work is properly cited.

Conventional electronics are typically fabricated on flat surfaces. However, real‐world objects are significantly 3D. It is therefore highly desirable to develop fabrication methods that can be deployed on the nonflat surfaces of 3D objects. Several methods have been used to achieve 3D electronics including capillary interaction involving liquid solder,^1^ thermopressure‐forming,^2^ rolling 2D electronic sheets into 3D,^3^ and controlled growth of nanostructures.^4^ Recently, significant progress has been made on the fabrication of 3D shapes using shape memory polymers, which undergo shape change in response to external stimulus, such as heat, light, electrical, or magnetic field.^5^ Examples include the fabrication of microfluidic channels,^6^ cell encapsulation containers,^7^, ^8^ drug delivery microcapsules,^8^, ^9^ 3D tissue scaffolds,^10^ and vascular stents.^11^ In addition, organic transistors have been fabricated onto shape memory polymer substrates for bio‐interface applications.^12^ However, it is still challenging to achieve 3D electronics with controlled shapes and to firmly wrap these devices around the contours of irregularly shaped objects.

Here, we describe a bilayer structure of shape memory polymers and polyimide for achieving shape‐controlled, self‐wrapped 3D single‐walled carbon nanotube (SWNT) electronics. This method involves the fabrication of flexible SWNT devices on a planar polyimide substrate followed by shape control using shape memory polystyrene on the back of the polyimide. SWNTs are chosen as our active semiconductors because they have high charge carrier mobility and high surface area for transistor and sensor applications.^13^, ^14^ They possess excellent mechanical flexibility and thermal stability required for our 3D fabrication process.^14^, ^15^ Finally, their solution processability is important for future low‐cost printing process.^16^, ^17^

We study the shape control of these bilayer structures by performing heating experiments combined with theoretical prediction of the resulting shape change. By heating the bilayer devices locally, we achieve multistep folding (development of curvature in various directions at different times) using the bilayer structure. In addition, the bilayer structure can be conformly wrapped to irregularly shaped objects. To test the compatibility of device properties with the 3D device fabrication, we bend flexible SWNT transistors on polyimide at a curvature of 750 μm and heat them at temperatures up to 220 °C; no changes in transistor behavior are observed. Devices (e.g., transistors, hydrogen sensors, temperature sensors, or memory devices) conform to various irregular objects are demonstrated based on SWNT thin film transistors (TFTs).

## 2 Results and Discussion

### 2.1 Inducing Controlled Curvature in Bilayer Freestanding Sheets with Heating

**Figure 1:**
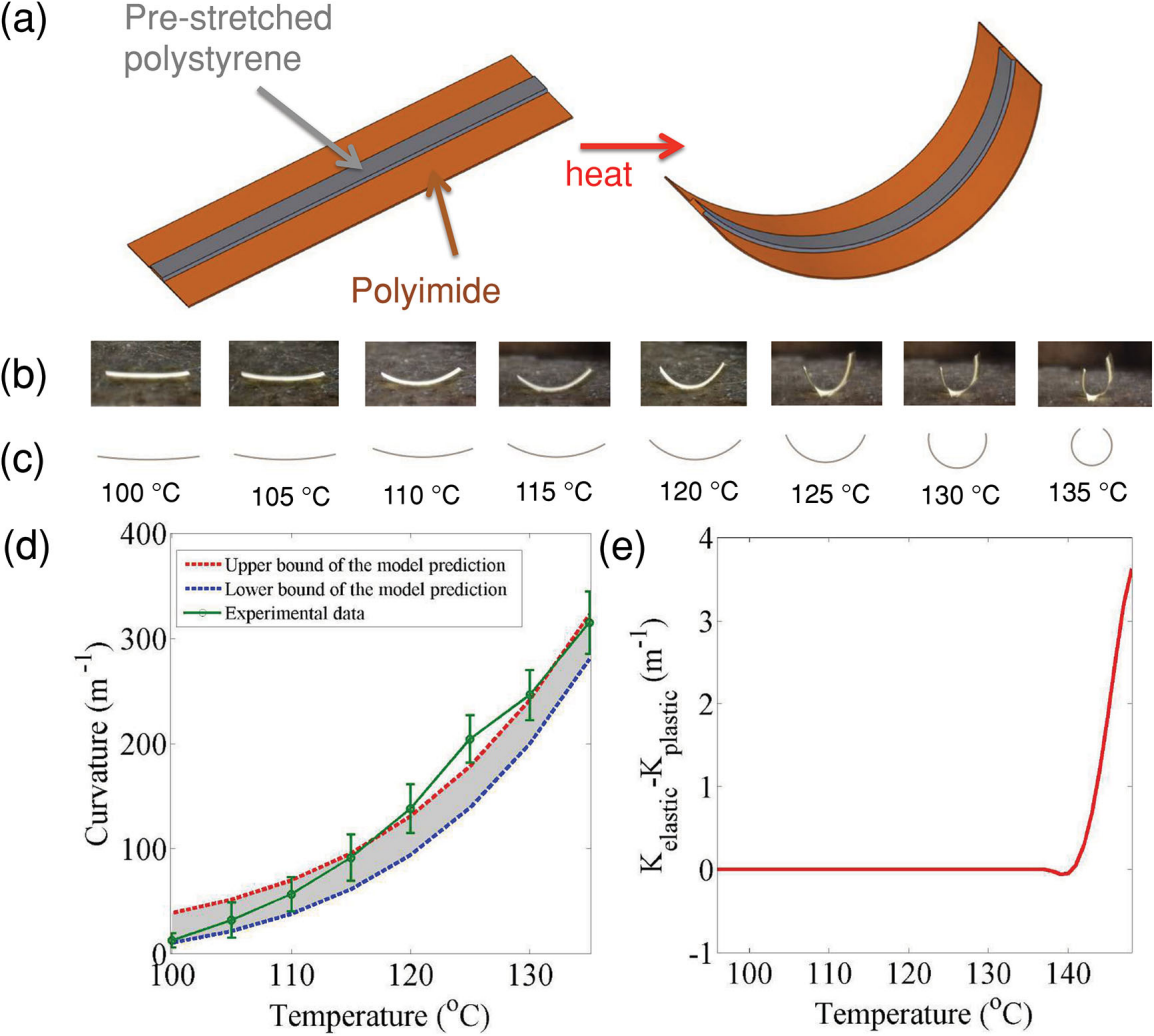
Predicting the curvature of bilayer structure by theoretical modeling at different temperatures. a) Schematic showing the shape changes of bilayer structure upon heating. b) Photographic images of the bilayer structure. c) Prediction of the curvature from our elastic model. d) Comparison of the radius of curvature between theoretical and experimental results as a function of temperature (upper bound and lower bound of the model are from the errors of input parameters from measurements). e) Curvature difference between elastic and plastic models as a function of temperature.

In our bilayer structure, a prestretched polystyrene shape memory polymer is chosen as the first layer because it undergoes a significant size reduction upon heating. Such films have previously been used for microfluidics^18^ and self‐folding boxes,^19^ but not demonstrated with electronic devices. Polyimide is chosen as the supporting substrate because it is thermally robust, solution‐processable, and can be used to fabricate ultrathin devices with ultrahigh mechanical flexibility. Actuation is driven by the entropically driven shrinkage of the prestretched polystyrene upon heating, which causes internal tensile stress in the polystyrene layer. As a result, the composite structure bend toward the polystyrene side. A schematic of induced curvature of using the bilayer structure is shown in **Figure** **1**a. As the temperature increases from 100 to 135 °C at a constant heating rate of 2 °C s^−1^, the curvature of the bilayer structure becomes higher (Figure 1b). To enable the design of programmable shapes, we first applied the Euler–Bernoulli beam theory to verify that the observed bending behavior can be understood in terms of the fundamental principles of mechanics. In this analysis, the assembly of the polyimide layer and polystyrene layer is treated as a composite beam. The polystyrene layer is assumed to experience a (negative) thermal strain. For simplicity, the only other strains we consider are the elastic strains in both layers. This analysis is subsequently generalized to allow plastic deformation in the polystyrene layer.

The analytic model predicts the shape of the bilayer and the stress distribution in the cross section (for details see Figure S1 and “derivation of plastic solution” in the Supporting Information). The predictions in the temperature range of 100 to 135 °C, when assuming thermal and elastic strain only, are summarized in Figure 1c and Figure S2, Supporting Information. From Figure S2, Supporting Information, we can observe clearly that the maximum stress always occurs at the interface, with the poly­styrene side in tension and the polyimide side in compression. The stress at every point in the cross section tends to increase with rising temperature, leading to an increasing curvature at higher temperatures. The shape change predicted by the model (shown in Figure 1c) is in good agreement with the experimental images in Figure 1b. A comparison of the predicted and measured curvature as a function of temperature is shown in Figure 1d. The error bars were determined from the largest and smallest curvatures from the experimental shapes in Figure 1b. In a more refined model, the polystyrene layer is allowed to undergo plastic deformation when the stress reaches its yield stress. However, this refinement leads to little change in the predicted curvature of the bilayer structure. The difference in the predicted curvature from the elastic‐only and elastic‐plastic models is zero at temperatures below 140 °C (because the yield stress is not yet reached) and remains small in the temperature range of 140–148 °C (Figure 1e, for details see “derivation of plastic solution” in the Supporting Information).

**Figure 2:**
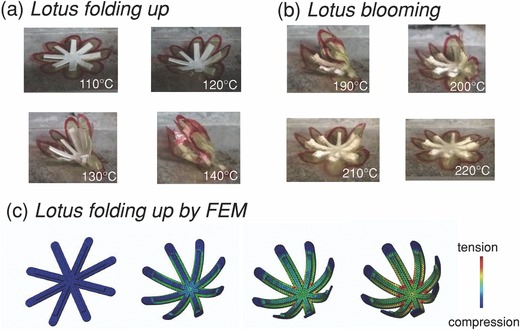
a) Lotus folding up process upon heating from 105 to 140 °C at a rate of 2 °C s^−1^. b) Lotus blooming process up further heating from 190 to 225 °C at a rate of 2 °C s^−1^. c) Schematic generated by finite element method (FEM) simulation showing the lotus folding‐up process.

Given the above understanding of our bilayer structure, we sought to use this bilayer architecture in complex experimental settings. We show an example of utilizing the bilayer structure by fabricating a foldable lotus flower. The prestretched polystyrene layer is patterned and attached to a flat, lotus‐shaped polyimide substrate. Folding is achieved by heating the flower petals from 105 to 140 °C at a rate of 2 °C s^−1^ (**Figure** **2**a). At 190 °C, the prestretched polystyrene starts to soften and therefore the flower starts to bloom (unfold) again. Until 225 °C (near the melting point of polystyrene), the flowers are fully extended as shown in Figure 2b. We also used the finite element method (FEM) to simulate the lotus folding process, which also predicts the internal stress distribution during the folding process of the flower (Figure 2c and Movie S1, Supporting Information). For simplicity, the physical dimensions of the structures in the FEM model do not exactly match those in the actual bilayer structure. This is due to the very large thickness difference between the polystyrene and polyimide layers. Using the real dimensions would require the use of an excessively large number of finite elements. Hence the FEM simulations are not meant to make quantitative predictions of the curvature‐temperature relation. Instead, they provide a qualitative description of the internal stress distribution during the folding process. The red region near the base of the polystyrene layer (Figure 2c) is in tension and the blue‐color area in the polyimide layer is in compression. This demonstration of fabricating shape‐controlled, freestanding objects with an electronics‐compatible bilayer structure shows the possibility of fabricating 3D electronic circuits or sensors that may be used in various shapes.

### 2.2 Creating 3D Shapes by Programmed Multistep Folding

**Figure 3:**
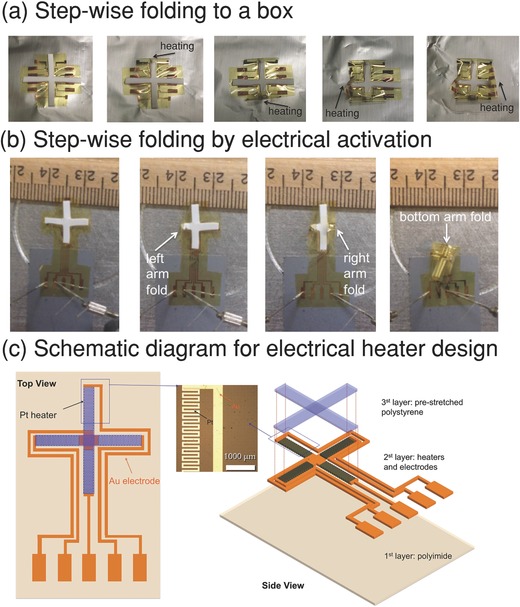
a) Fabrication of a box by step‐wise local heating of the bilayer structure (first graph) at the top arm (second graph), bottom arm (third graph), left arm (fourth graph), and right arm (fifth graph) in that sequence. b) Stepwise folding by electrical activation of a bilayer structure (first graph). Folding at the left arm (second graph), right arm (third graph), and bottom arm (fourth graph) in that sequence. c) Schematic diagram showing the electrical heater design.

In some applications, controlled sequential folding may be necessary. By locally heating a certain region of our bilayer structure, we can activate its folding in a step‐by‐step manner for achieving desired, complex shapes. Local heating can be applied by using a hot air gun. In **Figure** **3**a, we show an example of multistep folding of a box. Cross‐shaped prestretched polystyrene was attached to a polyimide film with devices (Figure 3a, left image). By applying local heating to each leg of the cross, we achieve folding from top, bottom, left, and right in sequence until the final box is fabricated.

The selective addressing by a hot air gun will only be possible if the objective structure is exposed and easily accessible. To achieve a more versatile platform that can fold into shapes even in an enclosed environment, we fabricated heaters and electrodes on the polyimide substrate for multistep folding by electrical activation. We chose platinum metal wires as the electrically addressable resistive heaters. The heating power generated from the resistive heater depends on the resistance of the metal wire and the applied voltage. Table S1 (Supporting Information) shows the required voltage for the Pt heaters to reach different temperatures. By applying 20 V to the heaters for several seconds, we can achieve a sufficient power for the bending of individual legs in sequence as shown in Figure 3b and Movies S2–S4, Supporting Information. The schematic showing how cross‐shaped prestretched polystyrene is attached to the electrodes with patterned heaters is presented in Figure 3c. This demonstration shows the opportunities for electrically controlled fabrication of 3D electronics.

### 2.3 Conformal Wrapping on Irregular‐Shaped Objects by Rapid Heating

**Figure 4:**
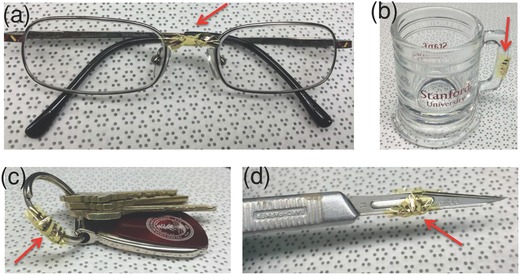
Conformal self‐wrapping of devices (pointed by red arrows) on different irregular objects. a) Eyeglasses. b) Shot glass. c) Key ring. d) Scalpel.

In addition to constructing preprogrammed shapes from the bilayer sheet by selective heating or electrically addressed thermal activation, the bilayer structure allows conformal wrapping of electronics onto any irregularly shaped objects by rapid heating (an example of self‐wrapping action is shown in Movie S5, Supporting Information). Examples of our self‐wrapped nanotube devices on 3D objects are shown in **Figure** **4**: including eyeglasses (Figure 4a), glass handle (Figure 4b), key ring (Figure 4c), and surgery tool (Figure 4d). The ability to attach electronics to common objects and tools could enable a variety of applications including: (1) Hydrogen gas sensing for fire safety with wrapped devices on eyeglasses or key rings; (2) body temperature or drink temperature sensing with wrapped temperature sensor on glass; (3) chemical sensing and sensing data storage (with memory device) during a surgery.

**Figure 5:**
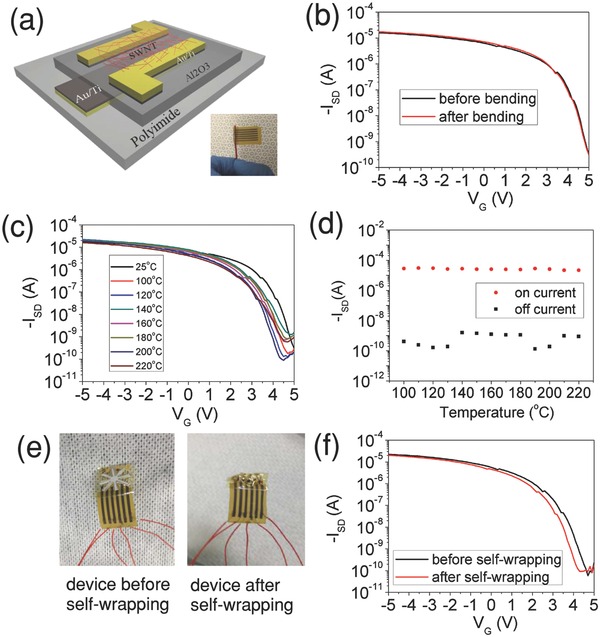
Effects of bending, temperature, and self‐wrapping on SWNT transistor characteristics: a) Schematic diagram of the device structure and bending of the device (inset: image of the bent device around a wire of 0.75 mm radius). b) Transistor characteristics of the device before and after bending at a 0.75 mm bending radius. c) Transistor characteristics of the device after heating to a temperature up to 220 °C with a bending radius of 0.75 mm (measurements were taken at room temperature after heating). d) On and off current of the device at different temperatures. e) Image of a SWNT transistor before and after self‐wrapping process. f) Transfer characteristics of the device before and after self‐wrapping process.

The electronic devices most compatible with the heat‐activated self‐wrapping process are carbon‐based electronics due to their high mechanical flexibility, thermal stability, and air stability. In this work, we used SWNT electronic devices for demonstration of this concept. It was demonstrated previously that flexible SWNT electronics could survive under mechanical deformation.^14^, ^20^, ^21^ However, 3D SWNT electronics with arbitrary shape has not been reported before. In order to prove that the SWNT devices can survive the self‐wrapping processes described above, we further tested the mechanical and thermal stability of SWNT devices produced on bilayer substrates. The SWNT devices are fabricated on polyimide substrates using our previously reported methods^22^ (see methods) before attachment of the prestretched polystyrene. We first severely bent the devices around a wire at a bending radius of 0.75 mm as shown in **Figure** **5**a. Since it is difficult to measure the device characteristics during severe bending, we attached a flexible polyimide/copper connector to the devices (see methods). As shown in Figure 5b, there is no change in device performance after bending. To test the thermal stability, we heated the bent devices at temperatures of 100, 120, 140, 160, 180, 200, and 220 °C, and measured their transfer characteristics after heating: No obvious change in device characteristics was observed (Figure 5c,d). In addition, we tested the transfer characteristics of the device before and after sever shape‐deforming process (Figure 5e,f) and also did not observe significant changes in device performance. Therefore, we conclude that SWNT transistors fabricated using our process can survive after the heating and deformation required for fabrication of 3D electronics. This is a result of good mechanical property of SWNTs and the use of thin polyimide substrate, which has been shown to enable fabrication of crumbled organic electronics previously.^23^

**Figure 6:**
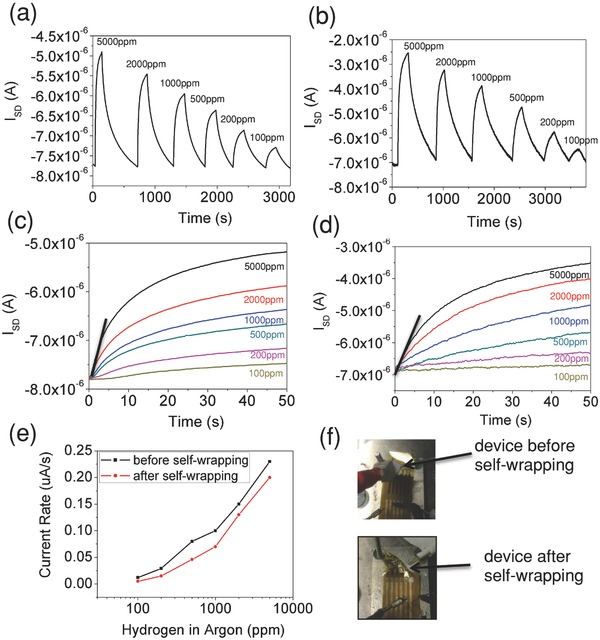
Hydrogen sensor device characteristics. Pd‐decorated SWNT sensor exposed to different concentration of hydrogen relative to argon a) before and b) after self‐wrapping process. The initial current rising rate of Pd‐decorated SWNT sensor exposed to different concentration of hydrogen gas relative to argon c) before self‐wrapping and d) after self‐wrapping. e) Plot of the current rising rate versus hydrogen concentration in argon before and after self‐wrapping process. f) Image of the device before and after self‐wrapping.

In addition to transistors, several other types of devices such as chemical sensors, temperature sensors, and memory devices are demonstrated on the self‐wrapped SWNT transistor‐based devices. A 2 nm layer of palladium was deposited on the SWNT network to enable selective sensing of hydrogen gas.^24^ It was reported that the dissolution of hydrogen on Pd lowers the work function of Pd and causes electron transfer from the Pd to the underlying SWNT layer, resulting a lower hole carrier density in the p‐type SWNTs.^24^ The sensitivity of the hydrogen sensor before and after self‐wrapping is demonstrated in **Figure** **6**a,b. The device provides a sensitivity of up to 100 ppm, comparable to the best‐reported SWNT‐based hydrogen gas sensors,^20^, ^25^ both before and after self‐wrapping process. Since it takes a rather long time to achieve saturated current after exposure of hydrogen, the initial current rise rate can be used to determine the concentration of hydrogen gas for fast detection (Figure 6c,d). The initial current rise rates are indeed similar for both pre and post self‐wrapped devices as shown in Figure 6e,f, showing the robustness of the devices. We also demonstrate temperature sensors and memory devices based on nanotube transistors after self‐wrapping process. For temperature sensors, a self‐wrapped transistor was used to measure temperature from −30 to 80 °C. A clear reduction in resistance with increasing temperature is observed (Figure S3a, Supporting Information) and a shift of threshold voltage towards positive voltages (Figure S3b, Supporting Information). The observation agrees well with the temperature‐dependent electrical measurements of semiconducting SWNT TFTs on the rigid substrate.^26^ Furthermore, the large‐hysteresis of SWNT‐based devices can also be utilized for memory devices based on charge trapping (Figure S4a, Supporting Information).^27^ We measured the retention time of a self‐wrapped SWNT device by charging it at 10 V (program) and −10 V (erase) after 1500 s (Figure S4b, Supporting Information): With an order of magnitude difference between on‐ and off‐states, the performance is comparable to other high‐performance SWNT memory devices with oxygen‐treatment^21^ or nanocrystal decoration.^28^ These SWNT‐based hydrogen sensors, temperature sensors, and memory devices can be easily incorporated in any of the irregular objects or controlled freestanding shapes demonstrated earlier in this work.

## 3 Conclusion

In summary, we developed shape‐controlled, self‐wrapped 3D carbon nanotube electronics. We achieve controlled curvature by constant rate heating of the bilayer structure. The stress distribution during heating can be predicted by analytical and finite element methods. We can also achieve step‐by‐step folding using local heating or electrically activated heating. Furthermore, these shape‐controllable devices can also adapt to any irregularly shaped objects by heating without degradation in device performance. Transistors, hydrogen sensors, temperature sensors, and memory devices with high performance were demonstrated after self‐wrapping, compatible with shape forming process. The fabrication of 3D electronics through the bilayer structure demonstrated in this work is not only compatible with SWNT electronics but also applicable to other flexible electronics systems. Further work might include the application of bidirectional shape memory polymer to achieve reversible shape‐memory effects for these electronic devices.^29^

## 4 Experimental Section

*Preparation of Sorted SWNT Solutions*: The SWNT dispersion was prepared via the following steps similar to our previously reported procedures:^17^ 1) 5 mg of HiPco SWNTs (Unidym Inc.) and 5 mg rr‐P3DDT (Sigma‐Aldrich Inc.) were mixed into 25 mL toluene. 2) The solution was sonicated using an ultrasonicator (Cole‐Parmer 750‐Watt Ultrasonic Processor) at an amplitude level of 70% for 30 min. 3) The solution was centrifuged for 1 h at 17 000 rpm. 4) The supernatant in the centrifuge tube was carefully extracted and placed into a separate vial to obtain a sorted SWNT solution.

*Fabrication of Flexible Transistors*: The flexible SWNT devices were fabricated using our previously reported methods.^22^ The patterned gate electrode consisting of 40 nm of Au and 5 nm of Ti was thermally evaporated onto the polyimide. The Ti layer was used to provide nucleation sites for 30 nm of Al_2_O_3_, which was deposited by atomic layer deposition (ALD) at 150 °C onto the gate. The 5 nm Ti and 40 nm Au source and drain electrodes were then patterned by a standard lithography process. The device was then soaked in a solution of rr‐P3DDT dispersed semiconducting SWNTs for 14 h and blow dried with N_2_ gas. The SWNTs outside the channel region were etched by oxygen plasma.

*Fabrication of the Bilayer Structure*: Polyimide solution (HD MicroSystems) was first spread over a 4 inch wafer at 500 rpm for 30 s and then allowed to rest undisturbed for 1 min. The wafer was then rotated at 1200 rpm for 1 min to form a film. Next, the wafer was annealed at 90 °C for 90 s and then at 115 °C for another 90 s. The wafer was then heated from room temperature to 350 °C at a ramp rate of 2 °C min^−1^ and held at 350 °C for 30 min. Patterns were printed by a commercial inkjet printer onto prestretched polystyrene paper and then cut by scissors. Super‐glue was used to adhere the patterned prestretched polystyrene paper to SWNT devices on the polyimide.

*Fabrication of Flexible Connectors*: In order to measure the devices after bending and self‐wrapping process, flexible connectors were fabricated onto the polyimide substrate. A copper coating was used on top of a flexible polyimide film as a substrate. Then a layer of wax was printed onto the copper surface using a commercial wax printer (Xerox Phaser) followed by etching in ferric chloride solution at 50 °C for about 5 min until the nonwaxed surface (exposed copper surface) was completely etched away. The wax layer was then removed by sonicating in acetone.

*Device Characterization*: Transistor, memory, and hydrogen sensing measurements were performed under ambient conditions at room temperature. Transistor and memory measurements were done with Keithley 4200 SC semiconductor analyzer. Hydrogen sensing experiments was performed in a home‐built setup and the device characteristics was measured with Keithley 2635A and Keithley 2400. The hydrogen concentration is controlled by varying the mixture of hydrogen to argon gas flow rate. The temperature sensor measurement was performed with Agilent semiconductor parameter analyzer in nitrogen atmosphere of controlled temperature.

*Analytic Model of Elastic Beam Bending*: The solutions of the curvature and stress distribution of a composite beam under thermal and elastic strains are known analytically and are given in the Supporting Information. The input parameters for the model are listed as follows. The polyimide layer had a width *w*_1_ of 1 cm, height *h*_1_ of 10 μm, and a temperature‐independent Young's modulus *E*_1_ of 2000 MPa. The polystyrene layer had a width *w*_2_ of 0.2 cm, height *h*_2_ of 0.2 mm, and modulus *E*_2_ as a function of temperature T in Celsius degree (−5.1 T + 780) MPa (fitted from experimental results). The thermal strain *e* ^*^ of the polystyrene layer is expressed as 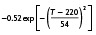 to fit the data from experimental measurements. The temperature range of the calculation is from 101 to 148 °C.

*Numerical Model of Plastic Beam Bending*: Here the polystyrene is modeled as an elastic‐perfectly plastic material (i.e., with no strain hardening). Once the plastic strain starts, analytic solutions to the curvature and stress distribution are no longer attainable, and the equations are solved numerically instead. The details of the equations and numerical algorithms are described in the Supporting Information. In addition to the parameters used in the elastic model (above), here the yield stress of polyimide *σ_y_* is also expressed as (70 − 0.73 T + 0.0018T^2^) MPa to fit our experimental measurements. Because plasticity is history dependent, a series of calculations are performed with the temperature increasing from 101 to 148 °C in steps of 1 °C. The solution from the previous temperature is used as the initial condition at the next temperature. A coupled set of equations are solved in Matlab using the fsolve function with the trust‐region‐dogleg algorithm.

*Measurement of Young's Modulus*: The Young's moduli of the polyimide and prestretched polystyrene layers were extracted from the linear elastic region of stress–strain curves measured on an Instron 5565 testing station. A gauge length of 10 mm was used for the polystyrene while a gauge length of 5 mm was used for the polyimide. In both cases the films were about 20 mm wide and a constant strain rate of 5 mm min^−1^ was used.

*Finite Element Method (FEM) Simulations*: The FEM simulations were performed using the commercial software ABAQUS. A double‐layer model with eight equal‐shaped arms that formed a shape of the union jack crosses was created to simulate the flower structure. The material type of the bottom layer is set to polyimide with the thickness of 1 mm, modulus of 2000 MPa, and Poisson's ratio of 0.3. The material type of the top layer is set to polystyrene with thickness of 2 mm, modulus of 300 MPa, Poisson's ratio of 0.35, the yield stress of 60 MPa, and the thermal expansion coefficient of −0.005 °C^−1^. The assembly was meshed with 10431 C3D4T elements for the type of coupled temperature‐displacement. A 5 × 5 mm^2^ patch at the center of the bottom surface was partitioned and fixed as the boundary condition. A predefined temperature field was applied to the whole structure to set the initial temperature of 100 °C. To fold the structure, a uniform body heat flux was applied and the geometric nonlinearity was considered during the heating process.

## Supporting information

As a service to our authors and readers, this journal provides supporting information supplied by the authors. Such materials are peer reviewed and may be re‐organized for online delivery, but are not copy‐edited or typeset. Technical support issues arising from supporting information (other than missing files) should be addressed to the authors.

Supplementary
