# Supplementary material for: Shape‐Controlled, Self‐Wrapped Carbon Nanotube 3D Electronics
Source: Adv Sci (Weinh). 2015 Jun 1;2(9):1500103. doi: 10.1002/advs.201500103 (PMC5115380; doi:10.1002/advs.201500103)
Supplement: Supplementary file 1 — Supplementary [file ADVS-2-0k-s001.pdf]

Copyright WILEY-VCH Verlag GmbH & Co. KGaA, 69469 Weinheim, Germany, 2015.

Supporting Information

for *Adv. Sci.*, DOI: 10.1002/advs. 201500103

Shape-Controlled, Self-Wrapped Carbon Nanotube 3D Electronics

*Huiliang Wang, Yanming Wang, Benjamin C.-K. Tee, Kwanpyo Kim, Jeffrey Lopez, Wei Cai, and Zhenan Bao**

Supporting Information

Shape-Controlled, Self-Wrapped Carbon Nanotube Three-Dimensional Electronics

By Huiliang Wang, Yanming Wang, Benjamin C.-K. Tee, Kwan-Pyo Kim, Jeffrey Lopez, Wei Cai, Zhenan Bao

Dr. H. Wang, Y. Wang, Prof. W. Cai
Department of Materials Science and Engineering, Stanford University,
496 Lomita Mall, Stanford, CA 94305

Dr. B.C.-K. Tee
Department of Electrical Engineering, Stanford University,
350 Serra Mall, Stanford, CA 94305

Dr. K. Kim, J. Lopez, Prof. Z. Bao
Department of Chemical Engineering, Stanford University,
443 Via Ortega, Stanford, CA 94305
E-mail: zbao@stanford.edu

Prof. W. Cai
Department of Mechanical Engineering, Stanford University,
440 Escondido Mall, Stanford, CA 94305

†Address correspondence to zbao@stanford.edu

KEYWORDS: carbon nanotubes, shape memory polymer, 3D electronics, shape-controlled, self-wrapped

Derivation of the elastic solution

Definition of the symbols:

E_1: the Young's modulus for the polyimide layer

E_2: the Young's modulus for the polystyrene layer

w_1: the width of the polyimide layer

w_2: the width of the polystyrene layer

h_1: the height of the polyimide layer

h_2: the height of the polystyrene layer

y: the position along vertical axis

e^*: thermal strain of the polystyrene polymer as a function of temperature T

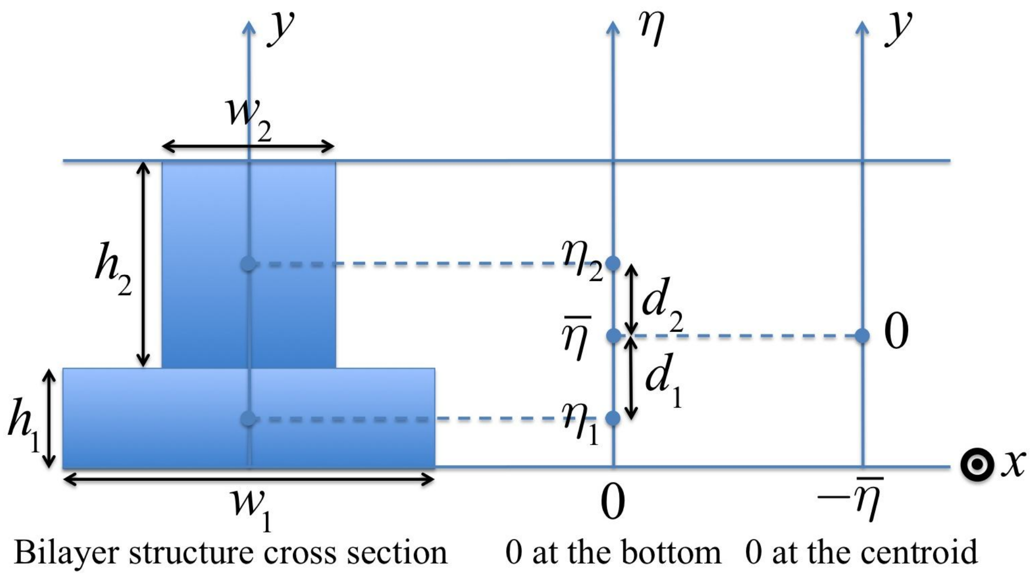

Figure S1 Schematic diagram explains the geometric parameters in the model.

This model treats the assembly of the polyimide film and the top pre-stretched polystyrene layer as a composite beam. The problem can be simplified to a one-dimensional bending problem assuming the length of the beam is much larger than its thickness and width. For simplicity, additionally we assume the system is purely in the linear elastic regime and any deformation due to shear across the section is neglected, so that the Euler-Bernoulli beam theory can be applied to obtain this analytical solution.

As set up in **Figure S1**, the coordinate system consists of a vertical *y*-axis and an *x*-axis perpendicular to the cross section plane. With the geometric parameters illustrated in **Figure S1**, $A_1 = h_1 w_1$ and $A_2 = h_2 w_2$ give the cross section areas of the polyimide and the polystyrene layers respectively. The centroid for the bottom polyimide layer η_1 is $\frac{h_1}{2}$ and η_2 for the top polystyrene layer is $h_1 + \frac{h_2}{2}$, setting the reference point at the bottom. Hence, the elastic centroid of the entire cross section of the composite beam is given by,

$$\bar{\eta} = \frac{E_1 A_1 \eta_1 + E_2 A_2 \eta_2}{E_1 A_1 + E_2 A_2} = \frac{E_1 h_1 w_1 \frac{h_1}{2} + E_1 h_1 w_1 (h_1 + \frac{h_2}{2})}{E_1 h_1 w_1 + E_2 h_2 w_2}. \tag{S1}$$

The beam's effective moment of inertia *(EIz)eff* is given by

$$(EI_z)_{eff} = E_1(I_1 + A_1 d_1^2) + E_2(I_2 + A_2 d_2^2), \tag{S2}$$

where $I_1 = \frac{w_1 h_1^3}{12}$; $I_2 = \frac{w_2 h_2^3}{12}$; $\mathrm{d}_1 = |\eta_1 - \bar{\eta}|$; $\mathrm{d}_2 = |\eta_2 - \bar{\eta}|$.

The bending of the bilayer system induced by the thermal strain can be understood by the superposition of two states. In state I, we can define a term of eigen-strain e^* that is the relative reduction in length at a given temperature *T* when the polystyrene layer is heated alone. For simplicity, we assume the thermal expansion of the polyimide layer is zero. When we heat the bilayer to temperature *T,* the pre-stretched polystyrene layer will shrink. Imagine at this time, an external tensile force *F* can be applied at the centroid of the polystyrene layer to stretch the polystyrene layer towards its original length before heating. A zero-strain state for the whole system can be reached at $F = E_2 \left| e^* \right| A_2$. The introduction of *F* will result in a non-zero stress in the polystyrene layer $\sigma_{xx}^{(2)} = E_2 \left| e^* \right|$. Meanwhile, the stress for the polyimide layer $\sigma_{xx}^{(1)}$ should still be 0.

In the actual system, the above-discussed external force *F* is not really applied, so this requires the superposition of state II on state I. In state II, we introduce a compression force $-F$ acts on the centroid of polystyrene layer in order to reach a force balance. Obviously, because this force does not act on the centroid of the composite beam, it generates a bending moment $M = F \cdot d_2$. If we set 0 of the *y*-axis at the centroid as shown in **Figure S1**, the normal strain perpendicular to the cross section plane is simply given by,

$$\varepsilon_{xx} = -\frac{M \cdot y}{(EI_Z)_{eff}} - \frac{F}{E_1 A_1 + E_2 A_2}. \quad \text{(S3)}$$

The stresses in the polyimide layer and polystyrene layers can be calculated using Hook's law neglecting other strain components:

$$\sigma_{xx}^{(1)} = E_1 \cdot \left(-\frac{M \cdot y}{(EI_Z)_{eff}} - \frac{F}{E_1A_1 + E_2A_2} \right), \tag{S4}$$

$$\sigma_{xx}^{(2)} = E_2 \cdot \left(-\frac{M \cdot y}{(EI_Z)_{eff}} - \frac{F}{E_1A_1 + E_2A_2} \right). \tag{S5}$$

Applying the superposition principle, the total stress in the polyimide layer is the sum of the stresses in state I and II,

for $0 - \bar{\eta} \le y \le h_1 - \bar{\eta}$

$$\sigma_{xx}^{(1)} = E_1 \cdot \left(-\frac{M \cdot y}{(EI_Z)_{eff}} - \frac{F}{E_1A_1 + E_2A_2} \right) = E_1 \cdot \left| e^* \right| \cdot \left(-\frac{E_2A_2 \left| \eta_2 - \bar{\eta} \right| y}{(EI_Z)_{eff}} - \frac{E_2A_2}{E_1A_1 + E_2A_2} \right), \tag{S6}$$

while the stress in the polystyrene layer is

for $h_1 - \bar{\eta} \le y \le h_1 + h_2 - \bar{\eta}$

$$\sigma_{xx}^{(2)} = E_2 \cdot \left(\left| e^* \right| - \frac{M \cdot y}{(EI_Z)_{eff}} - \frac{F}{E_1A_1 + E_2A_2} \right) = E_2 \cdot \left| e^* \right| \cdot \left(-\frac{E_2A_2 \left| \eta_2 - \bar{\eta} \right| y}{(EI_Z)_{eff}} + \frac{E_1A_1}{E_1A_1 + E_2A_2} \right). \tag{S7}$$

Finally, the curvature of the beam is given by

$$K = \frac{M}{(EI_z)_{eff}} = \frac{E_2 \left| e^* \right| A_2 \left| \eta_2 - \bar{\eta} \right|}{(EI_z)_{eff}}. \tag{S8}$$

Derivation of the plastic solution

Definition of the symbols:

ε^{tot} : total strain

e^{*}: thermal strain as a function of temperature

ε^{el} : elastic strain

ε^{pl}: plastic strain

y : the position along vertical axis

h_1 : the height of the polyimide layer

h_2 : the height of the polystyrene layer

K : the bending curvature

b : the strain at $y = 0$

E_1 : the Young's modulus for the polyimide layer

E_2 : the Young's modulus for the pre-stretched polystyrene layer

σ_Y : yield stress of the pre-stretched polystyrene layer

This model recognizes the bilayer system bending as a one-dimension plastic bending problem. We assume the plane sections remain planes during the entire bending process. Additionally, we assume the polystyrene to be perfectly plastic once yield occurs. The zero point of the vertical *y*-axis is set to the bottom of the beam in the analysis below. Starting with the compatibility condition, at every point in the bilayer system we should have

$$\varepsilon^{tot} = e^{*} + \varepsilon^{el} + \varepsilon^{pl} = Ky + b \ . \qquad \text{(S9)}$$

We assume the polyimide layer is always in the elastic region and its thermal expansion can be neglected, so the elastic strain is equal to the total strain for the polyimide layer.

$$\varepsilon^{el} = \varepsilon^{tot} \text{ for } y \le h_1 . \tag{S10}$$

For the pre-stretched polystyrene layer, the elastic strain is given by

$$\varepsilon^{el} = \varepsilon^{tot} - e^* - \varepsilon^{pl} \text{ for } h_1 < y \le h_1 + h_2 . \tag{S11}$$

The stress can be related to the elastic strain by multiplying the Young's modulus. Explicitly, the stress is given by

$$\sigma = \begin{cases} E_1(Ky+b) & y \le h_1 \\ E_2(Ky+b) - e^* - \varepsilon^{pl} & h_1 < y \le h_1 + h_2 \end{cases} . \tag{S12}$$

Since there is no external force applying onto the system, the total force and total bending moment should be 0, which leads to

$$\int_0^{h_1+h_2} \sigma \, dy = 0, \tag{S13}$$

$$\int_0^{h_1+h_2} \sigma \cdot y \, dy = 0 . \tag{S14}$$

Naturally, any increment of stress $\Delta\sigma$ should also obey these constraints:

$$\int_0^{h_1+h_2} \Delta\sigma \, dy = 0, \tag{S15}$$

$$\int_0^{h_1+h_2} \Delta\sigma \cdot y \, dy = 0 . \tag{S16}$$

Notice for the polystyrene layer, once yield occurs, σ is always equal to σ_Y since the polystyrene is assumed to be perfectly plastic.

An analytical solution for this problem is not attainable; in other words, the problem has to be solved numerically. We can discretize the [0, $h_1 + h_2$] interval on the y-axis into n

pieces so that the total force can be approximated by the sum of $\sigma(y_i)\Delta y$ from $i=1$ to $i=n$. In this way, a full solution of the system given at a certain temperature should include n stress $\sigma(y_i)$values from y_1 to y_n, the curvature K and a constant b. If we've already known the solution at one temperature T (for example, we can use the elastic solution derived in the previous section to find the solution for the temperature where the system is at the onset of yielding.), we are able to solve the system at the temperature $T+\Delta T$ by solving the following set of equations:

$$\sum_{i=1:n} \Delta\sigma(y_i)\Delta y = 0, \tag{S17}$$

$$\sum_{i=1:n} \Delta\sigma(y_i) y_i \Delta y = 0, \tag{S18}$$

$$\Delta\varepsilon^{pl}(y_i)\cdot\left(\sigma(y_i)+\Delta\sigma(y_i)-\sigma_Y\right)=0, \text{ for } i=1,2,\ldots,n. \tag{S19}$$

The above are $n+2$ equations, from which the $n+2$ unknowns (K, b and $\sigma(y_i)$ for $i=1, 2, ..., n$) can be solved. ($\Delta\varepsilon^{pl}$ is related to $\Delta\sigma(y_i)$ through Equation (S12), so it is not counted as an independent variable.) Equation (S19) indicates if the point y_i does not reach the yield point, the plastic strain is 0, which automatically balance the equation. On the other hand, if the yield occurs at position y_i, the plastic strain is no longer 0, which forces $\sigma(y_i)+\Delta\sigma(y_i)-\sigma_Y=0$ to satisfy the equation. Thus, we can solve out $\Delta\sigma(y_i)$ and the corresponding $\varepsilon^{pl}(y_i)$ using Equation (S12). Following the above steps, after the solutions at temperature $T+\Delta T$ are obtained, they can be used to solve for the next temperature state $T+2\Delta T$.

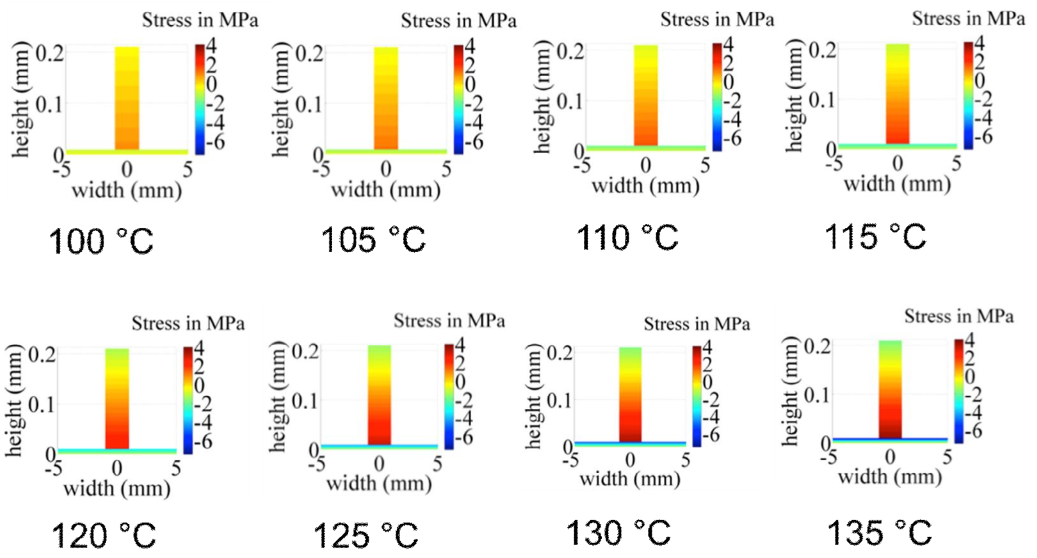

Figure S2 Stress distribution in the bilayer structure cross section from our elastic model. Top layer is pre-stretched polystyrene and bottom layer is polyimide.

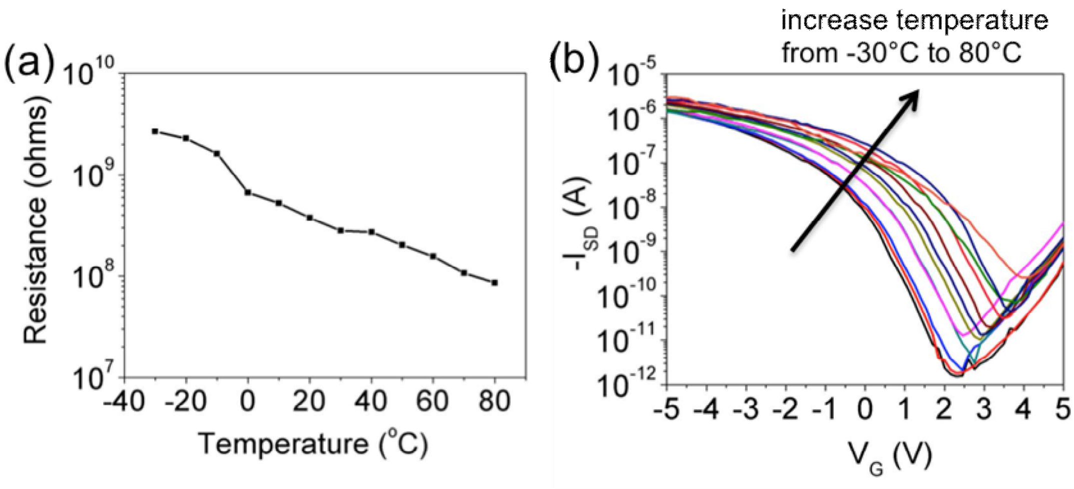

Figure S3 Self-wrapped SWNT transistor-based temperature sensor characteristics measured under Nitrogen. (a) Change in the resistance of the devices with change in temperature. (b) Change in transfer characteristics of the SWNT TFTs with increasing temperature (every 10 °C).

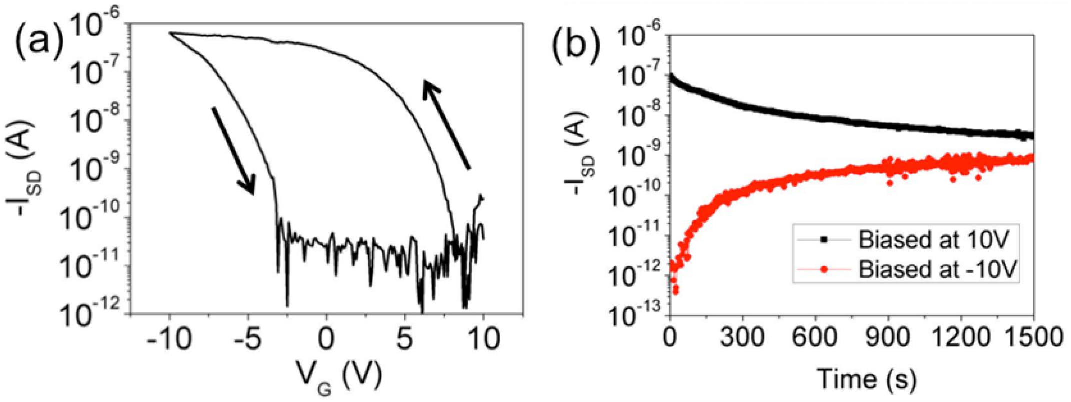

Figure S4 Self-wrapped SWNT transistor-based memory device characteristics. (a) Transfer characteristics showing the large hysteresis of the device. (b) Retention characteristics of SWNT transistor-based memory devices at programming and erasing gate voltages of 10 V and -10 V, respectively.

Voltage (V)	Current (A)	Power (mW)	Temperature (°C)
10	8.3×10^{-3}	69	70
15	10.7×10^{-3}	114	126
20	12.4×10^{-3}	154	184

Table S1. Temperature reached on different voltages applied to a platinum heater.
